# Optimal approaches for COVID-19 control: the use of vaccines and lockdowns across societal groups

**DOI:** 10.3389/fepid.2024.1308974

**Published:** 2024-07-09

**Authors:** Michael B. Bonsall, Chris Huntingford, Thomas Rawson

**Affiliations:** ^1^ Mathematical Ecology Research Group, Department of Biology, University of Oxford, Oxford, United Kingdom; ^2^ UK Centre for Ecology and Hydrology, Wallingford, United Kingdom; ^3^ Jameel Institute, School of Public Health, Imperial College London, London, United Kingdom

**Keywords:** COVID-19, optimal control, vaccination, non-pharmaceutical interventions, mathematical modelling, population cohorts

## Abstract

**Background:**

By March 2023, the COVID-19 illness had caused over 6.8 million deaths globally. Countries restricted disease spread through non-pharmaceutical interventions (NPIs; e.g. social distancing). More severe “lockdowns” were also required to manage disease spread. Although lockdowns effectively reduce virus transmission, they substantially disrupt economies and individual well-being. Fortunately, the availability of vaccines provides alternative approaches to manage disease spread. Yet, vaccination programs take several months to implement fully, require further time for individuals to develop immunity following inoculation, may not have complete coverage and/or may be imperfectly efficacious against the virus. Given these aspects of a vaccination programme, it is important to understand how NPIs (such as lockdowns) can be used in conjunction with vaccination to achieve public health goals.

**Methods:**

We use mathematical methods to, investigate optimal approaches for vaccination under varying lockdown lengths and/or severities to prevent COVID-19-related deaths exceeding critical thresholds.

**Results:**

We find that increases in vaccination rate cause a disproportionate decrease in the length and severity lockdowns to keep mortality levels below a critical threshold. With vaccination, severe lockdowns can further reduce infections by up to 89%. Notably, we include simple demographics, modelling three groups: vulnerable, front-line workers, and non-vulnerable. We investigate the sequence of vaccination. One counter-intuitive finding is that even though the vulnerable group is high risk, demographically, this is a small group and critically, per person, vaccination therefore occurs more slowly. Hence vaccinating this group first achieves limited gains in overall disease control.

**Discussion:**

Importantly, we conclude that improved disease control may be best achieved by vaccinating the non-vulnerable group coupled with longer and/or more severe NPIs.

## Introduction

The emergence of the SARS-CoV-2 virus in late 2019 has had devastating consequences across the globe, with all countries reporting levels of virus infection affecting public health responses. Since the emergence of this novel coronavirus, the policies implemented (in part using insights from previous pandemics) have focused on non-pharmaceutical interventions (NPIs). Such approaches included travel bans, limited household mixing, stay-at-home orders (“lockdowns”) and quarantines, social distancing and the closure and restriction of large mass gatherings. These NPIs require effective implementation, behavioural shifts and continued political and public support (1, 2) and have mitigated disease spread, levels of infection, rates of morbidity, and mortality (3–5). While these measures suppressed related COVID-19 deaths, NPIs have macroscale impacts on the economics of many nations (6–8), as well as, arguably, microscale implications, affecting the mental well-being of many individuals (9).

Although these NPI approaches can be succcessful in reducing virus spread, they are not a sustainable strategy, and other treatment-based approaches are required (10). Across different jurisdictions, several different vaccines were approved for safe use against COVID-19. However, efficacy levels vary from 70% to more than 90% (11).

Even if a vaccine works as intended, challenges remain; these are four-fold. The first is to instigate mass vaccination programs. The second is to prevent health services being overwhelmed while recognising that to vaccinate a nation takes time and that there is a lag of some weeks after inoculation before high immunity levels are achieved. The third issue is that many citizens could be reluctant to experience social and/or economic restrictions; persuading them to follow further restrictive rules, and especially for extended timeframes while vaccine programmes are completed, may be difficult. That is, many in society may be overly confident that the threat posed by the virus is reduced in light of the announcements of a successful vaccine, failing to recognise there remains a substantial time lag before sufficient levels of acquired immunity are realized. The fourth issue is that as vaccine roll-out increases, individuals may be more reluctant to take up a vaccine as disease prevalence levels fall, as herd immunity increases and as demand levels change.

There is therefore a requirement to determine the optimal balance between five factors. These factors are: (1) total number of deaths—this can be regarded as a proxy for healthcare capacity, (2) vaccination rate, (3) the level of lockdown restrictions impacting virus transmission rates, (4) the order in which different population groups are inoculated and (5) time spent in lockdown.

Here we solve aspects of this optimisation problem, based on predictions from a standard SIR (Susceptible, Infected and Recovered) model, modulated by a vaccination program. Specifically, we calculate for a range of prescribed total number of deaths, achievable vaccination rates of different groups and transmission rates, the optimal policy of vaccination delivery amongst population groups so as to minimise time spent in lockdown. A particular novel feature of our analysis is that we introduce heterogeneity in terms of group structure, with a focus on three main societal groups: vulnerable individuals, front-line worker, and non-vulnerable individuals. While arguably simplistic, this important classification of the population into three distinct groups provides a robust way to investigate optimal policy approaches that can be readily extended to be more complex (e.g. age-structured) groupings. Our overarching aim is to demonstrate how vaccination and lockdowns can, and should, be used together to achieve optimal disease control. In the the next section we introduce the mathematical frameworks and methods of analysis. In the results section, using numerical approaches and parameterised for the first wave of COVID-19 infection in the UK epidemic, we illustrate how different levels of vaccination, lockdown severity and prescribed maximum level of disease determine a minimal lockdown length for a derived optimal strategy of different vaccination rates across our three groups. In the discussion, we present our results in light of previous work on vaccination and non-pharmaceutical interventions.

## Mathematical models

### Basic unstructured model

Mathematical models such as the SIR (Susceptible, Infected, Recovered) models are commonly used frameworks to describe disease transmission (12). To explore the combined effects of vaccination and lockdowns on controlling infections, we begin by using a simple extension to an SIR framework in which vaccinated individuals (V) are accounted for separately from recovered (R) individuals. The dynamics for susceptible (S) individuals is such that the rate of change of the number of susceptibles decreases due to individuals becoming infected, is increased by any loss of immunity of people who have previously recovered, lowered by background death rate and as described here with the extended model, lowered as people are vaccinated. Hence, this gives for the time evolution of S:

(1) $$\frac{dS}{dt}=-\frac{\beta S(t)I(t)}{N(t)}+\sigma R(t)-\mu S(t)-\nu S(t).$$

The dynamics for infected individuals (I) are such that the number increases as susceptibles pass through an incubation period of length τ after which individuals become infectious. The number of infected decreases as people either die from COVID-19, die naturally, or recover. Hence I follows:

(2) $$\frac{dI}{dt}=\frac{\beta S(t-\tau )I(t-\tau )}{N(t-\tau )}\exp(-\mu \tau )-\left(\alpha +\mu +\gamma \right)I(t).$$

The dynamics for recovered individuals (R) are such that the number increases as people recover from infection, or decreases due to vaccination, non-COVID-19-related death, or loss of immunity, giving:

(3) $$\frac{dR}{dt}=\gamma I(t)-\left(\nu +\mu +\sigma \right)R(t).$$

Finally the dynamics for the total number of vaccinated individuals (V) is dependent on the vaccination rate, lowered only by a background death rate, and therefore there is an assumption of no immunity loss for those inoculated and no onward transmission of virus once individuals are vaccinated. Hence:

(4) $$\frac{dV}{dt}=\nu (R(t)+S(t))-\mu V(t).$$

In Equations 1–4, β is the disease transmission rate, σ the loss of immunity, μ is the background death rate, ν is the vaccination rate (acting on both susceptible and recovered individuals), τ is the incubation window (and exp(−μτ) is the survival rate through the incubation window), α is the disease induced death rate, and γ is the disease recovery rate. We introduce simulated lockdowns as a reduction in modelled disease transmission such that the value of β reduces to a range of values. The total number of people is N(t)=S(t)+I(t)+R(t)+V(t).

The optimal control problem (“Appendix”) is formulated in terms of finding an optimal vaccination rate (ν(t)) under different levels of lockdown restriction (i.e., different levels of β) within a time interval [0,T] so as to minimize “costs” of vaccination and disease-induced deaths above a threshold Z, as well as minimizing ongoing costs of infection (h[I(T),T]). Hence our overall quantity, J, that we wish to minimise by particular selection of time-evolving value of ν, satisfies:

(5) $$\begin{array}{rl} & \underset{0<\nu \leq 1}{\min(J[x,\;\nu ])}\\ & =\underset{0<\nu \leq 1}{\min}\left(h[I(T),\;T]+\int_{0}^{T}\left[\frac{\nu^{2}(t)}{2}V(t)+\exp(\alpha I(t)-Z)\rho \right]dt\right) \end{array}$$

with control inequality constraint 0≤ν≤1, subject to the system of governing differential equations ($x=[S,\;I,\;R,\;V{]}^{⊤}$) and initial conditions $x(t(0))=x_{0}$, where T is the length of epidemic and ρ is a scaling constant. The lockdown period is defined as L(T). The increasing quadratic “costs” associated with vaccination assumes that vaccination becomes increasingly difficult as the number of daily vaccinated individuals increases—this is one way to describe “vaccination demand” (see below for other approaches to this on the disease dynamics). The function exp(αI(t)−Z) describes the difference between the number of disease-induced deaths at time point t and a considered threshold value Z. Raising this difference to an exponent ensures that the contribution of (αI−Z) is small if αI<Z and that (αI−Z) contributes greatly to the cost functional J (Equation 5) when αI>Z. This means that deaths, above a certain threshold Z, are increasingly penalised. Threshold Z corresponds to exceeding an acceptable level of healthcare capacity. Here, ρ is a scaling constant that, within the objective functional, weights the relative contribution of the threshold mortality effects compared to vaccination “costs” on the minimisation. The function h[I(T),T]=αI(T) and represents the endemic costs of ongoing disease-induced mortality. The relative weighting of this terminal cost function against the costs of vaccination and cost of mortality exceeding a threshold within the epidemic (via parameter ρ), over time course T, can be used to evaluate minimizing costs associated with the immediate epidemic or those associated with the longer-term endemic costs of infection.

### Structured model

A key novelty of our analysis is that we extend the model to incorporate demographic structuring across the population. To represent this heterogeneity in population structure, we adopt a simplified approach. Rather than structuring by age group, we classify individuals into “front-line workers” (FR), “vulnerable” (VU) individuals or “non-vulnerable” (NV) individuals. This broad classification represents groups that experienced difference in the levels, and consequences, of SARS-CoV-2 infections. These differences occur through disease-induced levels of mortality, disease recovery rates and transmission rates (through the force of infection) (Tables 1, 2).

**Table 1:** Population-level parameters fixed across all groups. The units for all parameter values are scaled to per day.

Parameter	Definition	Value	Source(s)
β	Transmission rate	0.016	(13)
μ	Background mortality rate	$2.273\times 10^{-5}$	(14)
τ	Incubation time	5.1	(15, 16)
ν	Vaccination rate	0.001–0.05	This work
σ	Loss of immunity	(set to 0)	This work

**Table 2:** Population-level parameters specific to the different individual groups. The units for all parameter values are scaled to per day.

Parameter	Definition	Value	Source(s)
Front-line worker group			
α	Disease-induced death rate	0.0032	(17)
γ	Recovery rate	∼0.1	(18, 19)
Vulnerable individual group			
α	Disease-induced death rate	0.064	(17)
γ	Recovery rate	∼0.06	(19, 20)
Non-vulnerable individual group			
α	Disease-induced death rate	0.0032	(17)
γ	Recovery rate	∼0.1	(18, 19)

Transmission could then occur differently within and between groups, and we define the matrix $\beta_{\;j,i}$ as the probability that an infected individual in group i infects a susceptible individual in group j. The force of infection (the *per capita* rate of infection) on the $j^{\mathrm{th}}$ group is then:

(6) $$\chi_{j}=\sum_{i=1}^{3}\beta_{\;j,i}\frac{I_{i}}{N_{i}},\;$$

where $\frac{I_{i}}{N_{i}}$ is the frequency-dependent transmission function from the $i^{\mathrm{th}}$ group. The expected probability that a susceptible individual (in group j) acquires an infection, from any source, is then the sum of the products of transmission rate and proportion of infected individuals in the $i^{\mathrm{th}}$ group, for all i.

The use of Equation (6) allows us to retain the mathematical structure of the epidemiological dynamics of (1)–(4), now applied to the $i^{\mathrm{th}}$ group as: ?>

(7) $$\frac{dS_{i}}{dt}=-\chi_{i}S_{i}(t)+\sigma R_{i}(t)-\mu S_{i}(t)-\nu_{i}S_{i}(t),\;$$

(8) $$\frac{dI_{i}}{dt}=\chi_{i}(t-\tau )S_{i}(t-\tau )\exp(-\mu \tau )-\left(\alpha_{i}+\mu +\gamma_{i}\right)I_{i}(t),\;$$

(9) $$\frac{dR_{i}}{dt}=\gamma_{i}I_{i}(t)-R_{i}(t)\left(\nu_{i}+\mu +\sigma \right),\;$$

(10) $$\frac{dV_{i}}{dt}=\nu_{i}\left(R_{i}(t)+S_{i}(t)\right)-\mu V_{i}(t),\;$$

where the force of infection ($\chi_{i}$), vaccination rate ($\nu_{i}$), disease-induced death rate ($\alpha_{i}$) and recovery rate ($\gamma_{i}$) are group-specific parameters. The rate of loss of immunity (σ), background death rate (μ), and virus incubation time (τ) are population-level parameters, independent of group structure. For each group $N_{i}(t)=S_{i}(t)+I_{i}(t)+R(t)+V_{i}(t)$.

The optimal control approach introduced above can be extended to multiple groups. For the structured model, the objective functional (Equation 5) is now:

(11) $$\begin{array}{rl} & \underset{0<\nu \leq 1}{\min(J[x,\;\nu ])}=\underset{0<\nu \leq 1}{\min}(\sum_{i=1}^{3}h[I_{i}(T),T]\\ & \;+\int_{0}^{T}\sum_{i=1}^{3}\left[\frac{\nu_{i}^{2}(t)}{2}V_{i}(t)+\exp(\alpha_{i}I_{i}(t)-Z_{i})\rho \right]dt) \end{array}$$

where, as before, the terms on the right hand side represent terminal conditions/costs for ongoing infection in each group ($h[I_{i}(T),\;T]$), the “costs” of vaccination for each group ($\frac{\nu_{i}^{2}(t)}{2}V_{i}(t)$) and the mortality thresholds that are not to be exceeded for each group ($\exp(\alpha_{i}I_{i}(t)-Z_{i})\rho$). ν is a set of vaccination rates (ν=$\nu_{1}$,$\nu_{2}$,$\nu_{3}$) and the subscript i denotes group-specific state variable (number of infected and vaccinated individuals in the $i^{th}$ group, $I_{i}$ and $V_{i}$, respectively) or parameter (vaccination rate; $\nu_{i}$, disease-induced death rate $\alpha_{i}$, critical mortality level $Z_{i}$). The aim is to minimize the quantity (J[x,ν]) subject to the governing differential equations for the group-structured epidemiological dynamics ($x={S_{i},\;I_{i},\;R_{i},\;V_{i}}^{⊤}$)—Equations 7–10), a set of initial conditions and boundary constraints (see “Appendix” for further details).

### Optimal vaccination

To investigate the optimal vaccination rate under different levels of lockdown severity, and in order to keep disease-induced mortality below a critical threshold, we solve the optimal control problem defined by Equation 5 for the unstructured model (Equations 1–4) or Equation 11 for the structured model (three sets of Equations 7–10). Solutions for the optimal vaccination rate(s) are found following Pontryagin’s maximum principle (21). Using the objective functional (Equations 5 or 11), derived adjoint equations, and control inequality constraint (22), optimal dynamic vaccination rates (ν(t)) are calculated for varying prescribed levels of reduced disease transmission (β). We use a modified Runge-Kutta method [a forward-backward-sweep algorithm (21)] to find the optimal outcome. The full details of the appropriate Hamiltonian operators and adjoint equations used to identify the optimum vaccination rates are given in the “Appendix.”

### Vaccination, different groups and lockdowns

We also investigate the interplay between vaccination strategies amongst population groups for lockdowns of different prescribed lengths (L(T)). In doing this, we can investigate the optimal suppression of the virus (in terms of disease-induced mortality) for a variety of considered lockdown lengths and effectiveness, while keeping cumulative mortalities below a critical threshold. To do so we use the structured model (Equations 7–10). This model framework accounts for the three different societal groups, front-line workers (FR), a vulnerable (VU) group and a non-vulnerable (NV) group, and was solved numerically over 150 days. The model was parameterized with parameters estimated to be appropriate to the early epidemic wave in the UK (Tables 1, 2). We calculate the optimum vaccination strategies across different population groups. Hence, our main overall objective is to determine the optimal vaccine rate, for prescribed reductions in disease transmission and for different lockdown times, all calculated for different sequences of societal group vaccination. We can then determine if, for the same optimal vaccination rate, different sequences of vaccination can substantially lower lockdown length.

### Optimal lockdown times for different group order of vaccination

Importantly, we investigate the hypothesis that lockdowns can be used to support, in parallel, on-going vaccination programs in order to prevent infection rates crossing key thresholds. In particular, we consider how different strategies concerning the order in which groups vulnerable, front-line workers, and non-vulnerable are inoculated, for the same maximum infection rate thresholds, impacts on length of lockdown. Hence the optimum we search for is the shortest lockdown strategies for each threshold. Again, we solve the structured epidemiology model (Equations 7–10) numerically for different sequences of vaccination over 150 days. The chosen sequence is to deliver the vaccine to the first group for 30 days, followed by the first and second group from 31–60 days and all groups after 60 days. Critically, the vaccination rates, expressed as a fraction of the group vaccinated per day, are common to each group. This implies that as the vulnerable group is smaller, the number of people vaccinated per day is smaller, likely reflecting the actual situation. The optimal outcome in terms of group order vaccination strategy to achieve the shortest lockdown times, while maintaining cumulative mortalities below a critical threshold, is also determined for varying lockdown severities (i.e., levels of transmission reduction).

### Vaccination demand: declining vaccine uptake rates

A further issue that we consider is vaccination elasticity. Elasticity is used to describe how supply and demand varies given changes in (usually) the price of a commodity (23). In epidemiology, this idea of elasticity in demand has instead been linked to vaccine uptake and disease prevalence (24). With inelastic demands for vaccination, as disease prevalence falls and assuming that vaccination levels can be maintained, then the public health benefits of continued vaccination (and the resulting herd immunity) can be maintained as infection levels fall. However, if vaccine uptake is elastic with respect to disease prevalence, such that as disease prevalence falls individuals are less likely to take a vaccine, then the public health control of infections can be more challenging. Elasticity can prevent achieving the vaccination levels needed to reach the critical threshold for herd immunity.

Here we investigate the role of vaccine demand elasticity, via the time evolving vaccination rate, ν(t), on disease outcomes (in terms of cumulative levels of mortality) under different lockdown lengths and severities. Our working hypothesis, that we test, is that lockdowns can be used to offset vaccine elasticities to maintain and/or achieve virus control. We use numerical approaches to solve the full structured model over time (150 days) to find the optimal overall vaccination level, lockdown duration and severities that keep cumulative mortality below a critical threshold.

The code used for all numerical analyses and simulations is available at https://osf.io/xvunt/.

## Results

### Optimal vaccination

Solving the constrained optimisation problem (“Appendix”) shows that the optimal vaccination strategy is a function of the ratio between the susceptibles, recovered and vaccinated individuals:

$$\nu_{i}^{*}=\frac{\lambda_{1i}S_{i}+\lambda_{3i}R_{i}-\lambda_{4i}(R_{i}+S_{i})}{V_{i}}$$

where subscript i denotes the group (for the unstructured model i=1, for the structured model i=1,2,3), $\lambda_{\mathrm{yi}}$ are adjoint (Lagrange) multiplier variables associated with the state variables ($S_{i}$, $R_{i}$ and $V_{i}$). While the state variables constrain the minimisation of the objective functionals (Equations 5, 11), these multipliers can be thought of as representing costs of violating the state variable constraints (“Appendix”).

**Figure 1:**
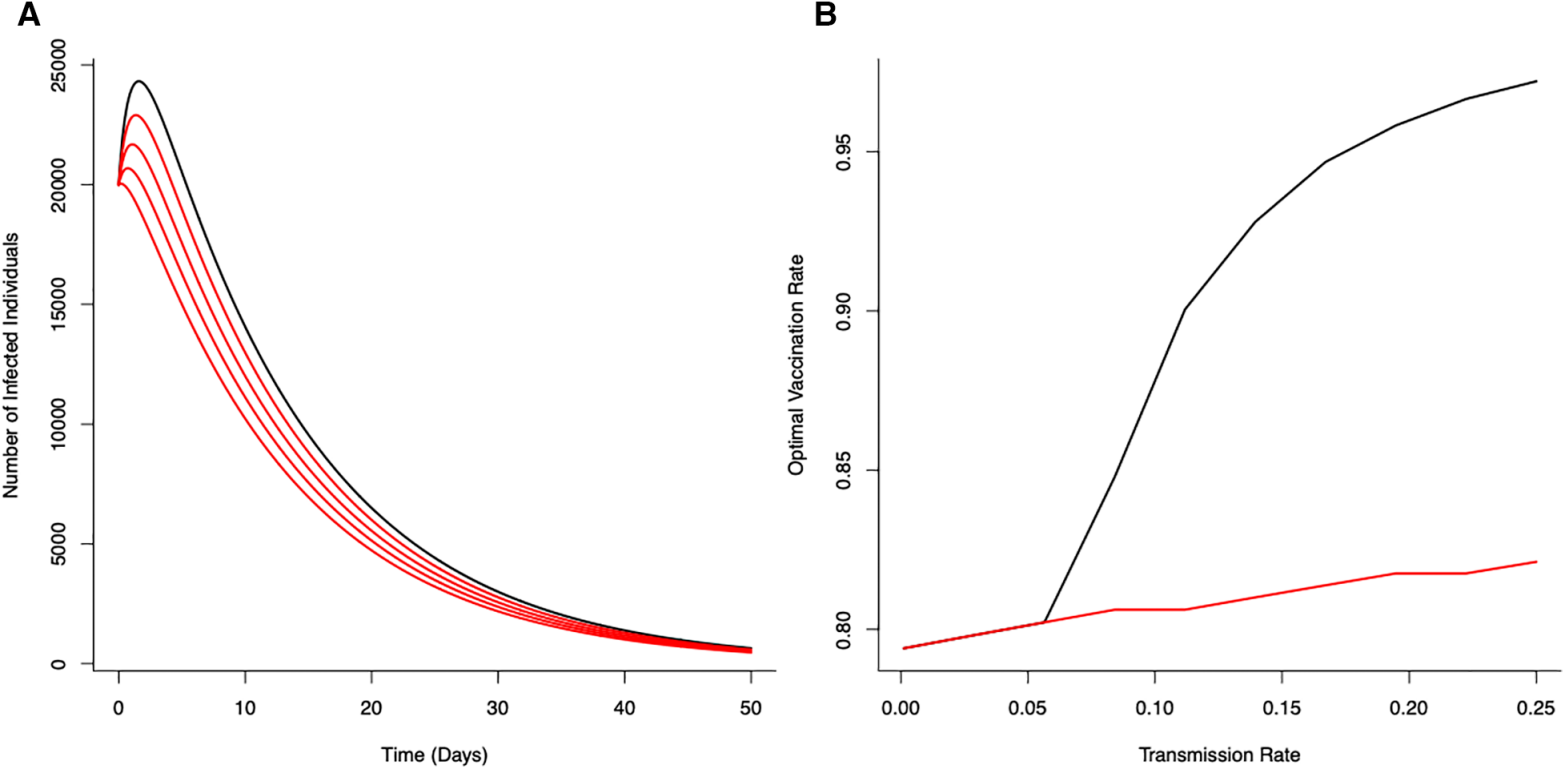
Optimal vaccination rate $\nu^{*}$ on disease control (from the unstructured S-I-R-V model) under a fixed critical threshold (Z) that instantaneous disease-induced deaths should not exceed. (**A**) The effects of reducing transmission (β) on disease dynamics for no reduction in transmission (β=0.25—black line) with progressive reductions (20%, 40%, 60% and 80%) in transmission shown in red (Z=100). (**B**) Optimal vaccination rate for different transmission rates for different fixed critical threshold (Z) (black line Z=1.0; red line Z=100). Optimal vaccination rate with low transmission (severe lockdown) reduces the potential for epidemic disease epidemics and minimize number of deaths. (Initial conditions $S(0)=5.8\times 10^{7}$, $I(0)=2.0\times 10^{4}$, R(0)=V(0)=0.0; Terminal conditions I(T)=100; Other parameters: β=0.25. α=0.0032, γ=0.1, μ=1/80, ρ=1.0).

Solutions for the optimal vaccination rate, $\nu =\nu^{*}$, are shown in Figure 1. Coupled with the threshold mortality condition, an optimal vaccination strategy can suppress the epidemic. However, for increasing lockdown severity (20%–80% reduction in transmission i.e., β), this can lead to the infection fading out without the characteristic epidemic growth curve associated with general S-I-R type dynamics.

With a canonical parameter set for the unstructured epidemiological dynamics (Figure 1), optimal vaccination strategies that keep daily disease-induced mortality below a critical threshold (Z=100) can reduce peak numbers of infections by ∼74% (with just vaccination) through to ∼89% (with vaccination coupled with a 80% reduction in transmission rate).

The (optimal) vaccination strategy is influenced by the stringency of the mortality threshold (Z) (Figure 1B). As might be expected, a more stringent threshold (i.e., allowing fewer deaths) necessitates higher levels of vaccination to achieve the expected level of control to ensure disease-induced mortality remains below that critical threshold. As also expected, decreasing potential disease transmission (e.g., through the use of NPIs) offsets the need for high levels of vaccination to achieve the necessary levels of disease control (Figure 1B). Notable is the strong non-linearity in Figure 1B; where, as a result of reductions in transmission (i.e, effects of different lockdown scenarios), the optimal vaccination rate declines in different ways for different critical thresholds.

**Figure 2:**
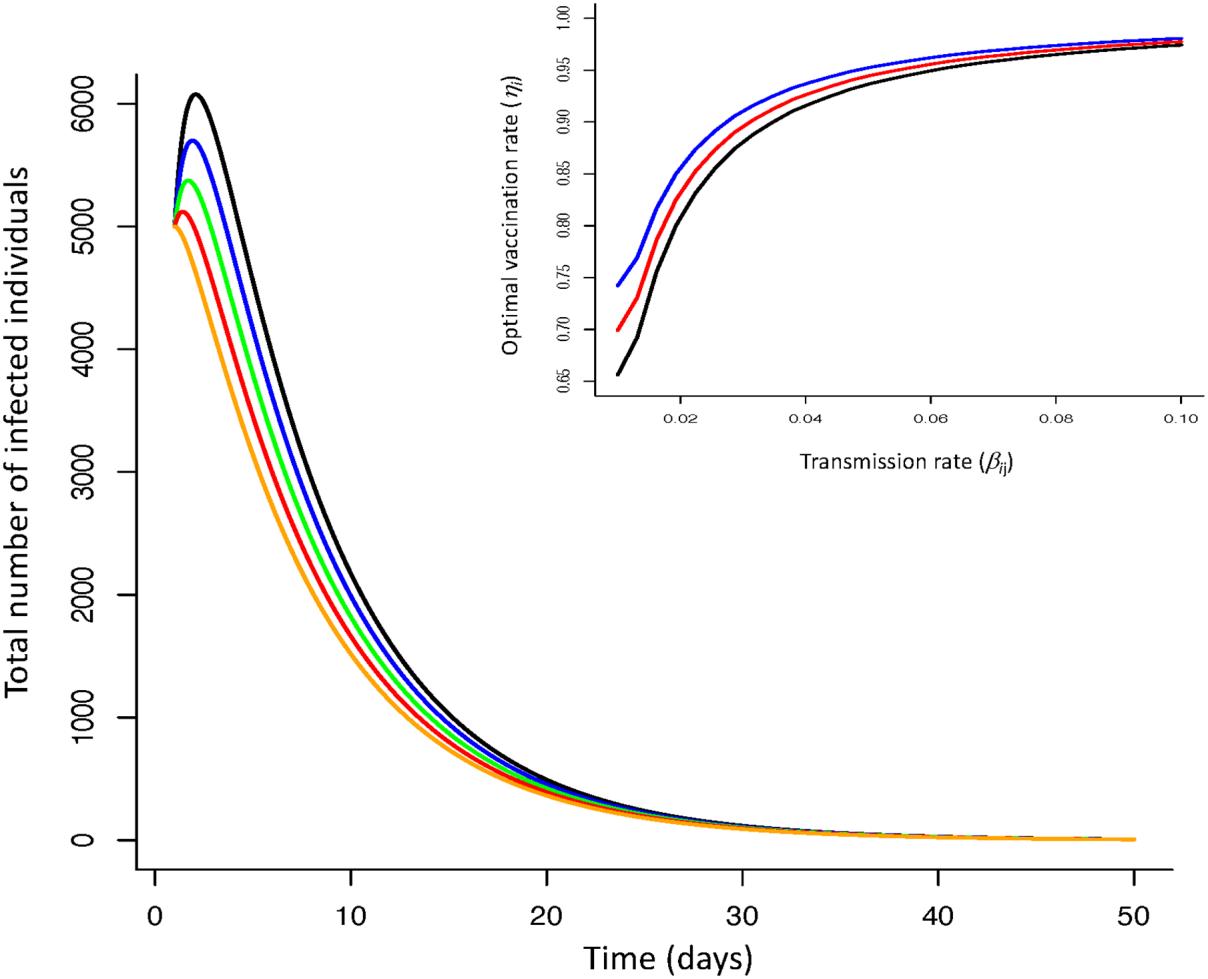
Infection dynamics for optimal vaccine dynamics across three groups (non-vulnerable, front line workers, vulnerable) for increasing severity of lockdowns (reductions in transmission rate; no reduction—black line; 20% reduction—blue line; 40% reduction—green line; 60% reduction—red line; 80% reduction—orange line). *Insert figure*—optimal vaccination rate as a function of transmission rate for vulnerable (blue line), front-line worker (red line), non-vulnerable group (black line). Increasing the severity of lockdowns (reduced virus transmission rate within and between groups) reduces infection dynamics when coupled with vaccination. More severe lockdowns (greater reductions in transmission) require lower vaccination rates to ensure mortality levels do not exceed a critical threshold. The optimal vaccine strategy is independent of lockdown severity but requires differentiation of this strategy between groups. [Parameters: transmission range: $\beta_{\mathrm{FR}}=\beta_{\mathrm{NV}}=0.01,\;0.1$, $\beta_{\mathrm{VU}}=0.001,\;0.1$; disease induced death rate: $\alpha_{\mathrm{FR}}=\alpha_{\mathrm{NV}}=0.032$, $\alpha_{\mathrm{VU}}=0.064$; recovery rate: $\gamma_{\mathrm{FR}}=\gamma_{\mathrm{NV}}=0.2$, $\gamma_{\mathrm{VU}}=0.1$; Initial conditions: $S_{\mathrm{NV}}=5.4\times 10^{7}$, $I_{\mathrm{NV}}=1000$; $S_{\mathrm{FR}}=1.0\times 10^{7}$, $I_{\mathrm{FR}}=2000$; $S_{\mathrm{VU}}=4.0\times 10^{6}$, $I_{\mathrm{VU}}=4000$; $\nu_{\mathrm{NV}}=0.3$; $\nu_{\mathrm{FR}}=0.4$; $\nu_{\mathrm{VU}}=0.5$. Terminal conditions: $I_{i}=100$; $Z_{\mathrm{NV}}=Z_{\mathrm{FR}}=Z_{VU}=1.0$, μ=1/80].

The optimal vaccination strategy can also vary between groups (Figure 2) depending on underlying epidemiological (demographic) dynamics associated with different groups. With a vulnerable class of individuals, more susceptible to disease, less likely to recover and more likely to suffer serious illness, decisions to focus initial vaccination on this group (for a given threshold of mortality) can affect optimal vaccination rates. Under no-lockdown scenarios, high vaccination rates ($\nu_{i}\approx 0.95$) for all groups and particularly the vulnerable group are required to ensure critical mortality thresholds are not exceed. However, as lockdown severity increases ($\beta_{\mathrm{ij}}$ are reduced), vaccination rates across groups can be lower leading to lower total number of infections over time (Figure 2).

### Vaccination, different groups and lockdowns

**Figure 3:**
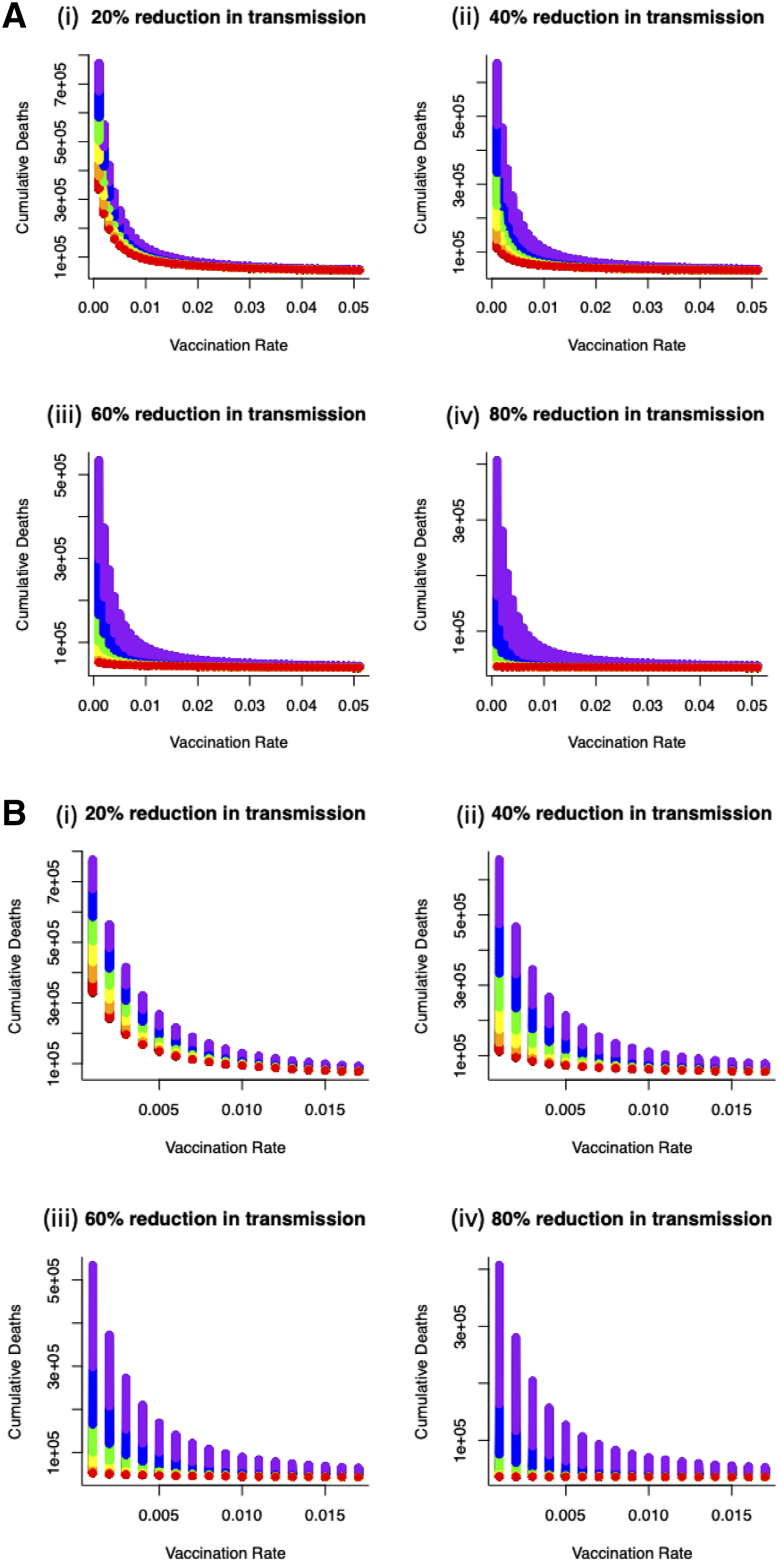
(**A**) Combined effects of vaccination and lockdown durations (rainbow colours blue/purple–short (15 days) through to red–long (95 days)) for different lockdown severities (where transmission is reduced by (i) 20%, (ii) 40%, (iii) 60% and (iv) 80%) on cumulative deaths. (**B**) For vaccination to be at all effective in reducing cumulative mortality (say ∼50K over 150 days) in the range of coverage anticipated (0.006–0.0152) then lockdown severity needs to reduce transmission by at least 60% or lockdown durations need to be unduly long (i) 20%, (ii) 40%, (iii) 60% and (iv) 80% reduction in transmission).

We now turn to optimal outcomes for the structured epidemiological dynamics (Equations 7–10), cumulative levels of disease-induced mortality is influenced by the length of time in, and severity of, lockdown along with the vaccination rate (Figure 3). Increasing the vaccination rate decreases the need for longer lockdowns and, under high vaccine coverage, the necessity for these lockdowns. While still essential, lockdowns can be of short duration when partnered with a vaccination program. This coupling of vaccinations and lockdowns can limit cumulative mortality levels. Under lockdowns where the level of transmission is only reduced by 20% or 40%, cumulative mortalities due to the virus are expected to be excessively high unless vaccine coverage is also high. Under more severe measures during lockdowns where transmission is reduced by 60% or 80% (Figure 3), shorter lockdowns can be sufficient to limit disease-induced mortalities.

**Figure 4:**
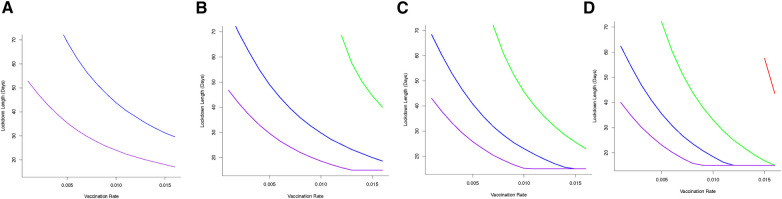
Optimal combination of vaccination rates (covering an expected vaccination rate range of 0.006–0.0152) and length of lockdowns to (under different severity to keep cumulative deaths below (**A**) 50 K (**B**) 60 K (**C**) 70 K or (**D**) 80 K for different lockdown severity (purple—80% reduction in β, blue—60% reduction in β, green—40% reduction in β, red—20% reduction in β). From (**A**)—50 K mortality threshold, for a weakly efficacious vaccination rate (0.005), depending on the severity of lockdown, lockdown durations could be 30 days (for 80% reduction in beta) or 70 days (for 60% reduction in beta). At this level of vaccination (0.005) and for a lockdown where β is only reduced by 20% or 40% it is not feasible to keep cumulative mortality below the 50 K threshold.

More specifically, for vaccination to be effective in reducing cumulative mortality (set here at ∼50,000 over 150 days), and in the range of anticipated vaccination rate (0.006–0.0152), then lockdown severity needs to reduce transmission by at least 60% or lockdowns need to be unacceptably long and extend for more than 90 days. Furthermore, these sort of lockdowns can be used to offset weakly efficacious vaccine rates to ensure that disease-related mortalities are kept below the (nominal) threshold of 50,000 (Figure 4).

**Figure 5:**
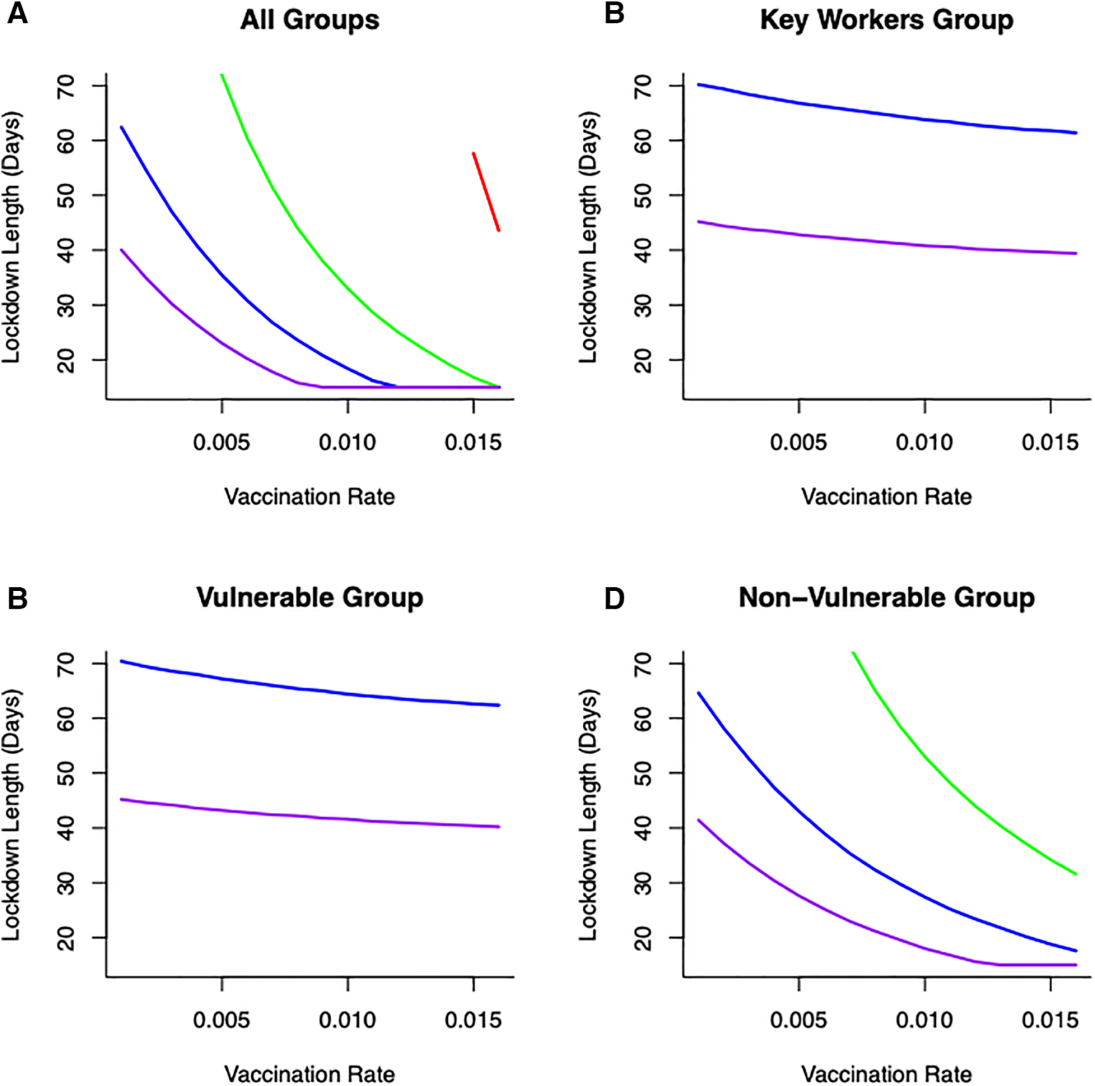
Combinations of vaccination across different groups: (**A**) all groups vaccinated (**B**) only key worker group vaccinated (**C**) only vulnerable group vaccinated (**D**) only non-vulnerable group vaccinated, lockdown duration (in days) and lockdown severities (in terms of reducing transmission—[purple—80% reduction in β, blue—60% reduction in β, green—40% reduction in β, red—20% reduction in β)] on keeping cumulative mortality less than 80K. Vaccination across the non-vulnerable group provides greater opportunities to keep mortality below critical threshold with lockdowns of short duration and/or less restrictive.

To understand more fully vaccine delivery strategies between different parts of society, we begin by determining how focusing initial vaccine delivery on *a single group* affects the likelihood of keeping levels of mortality below the (optimal) threshold. If vaccines are delivered singly to vulnerable or key worker groups, then lockdowns would still be necessary and they would need to be reasonably severe (>60% reduction in transmission) to reduce cumulative mortality below key thresholds (Figure 5). Notable is that less severe lockdowns when vaccinating either of these two groups are not sufficient to keep cumulative mortality below a critical threshold. This key finding is critical, and requires consideration in light of the decision that many countries took to vaccinate the most vulnerable first. The reason for this finding is due to the demographic differences between these two groups (where population sizes in these groups are relatively small) compared to the non-vulnerable group (which contains the majority of the population). For example, consider a vulnerable group of 500,000 people, and a non-vulnerable group of 10,000,000 people. If we vaccinate roughly 5% of each group each day this would lead to vaccinating either 25,000 vulnerable people, or 500,000 non-vulnerable people, daily. Hence, even if individuals in a vulnerable group are an order of magnitude more likely to die of COVID-19, more lives would be saved by vaccinating the 500,000 non-vulnerable group. For some scenarios, this might be likely, if non-vulnerable people can be vaccinated (in units of people per day) at a rate that is an order of magnitude larger, for instance through the creation of mass vaccination centres. In those circumstances, a more robust approach to keeping mortality below the critical threshold would be to vaccinate across the non-vulnerable (*NV*) group first and gain from the related shorter and/or less severe lockdown (Figure 5).

### Optimal lockdown times

Optimal outcomes, in terms of shortest possible lockdown times based on order of vaccination between groups (rather than by solution to Equation 11), are shown in Figure 5 for different transmission reductions. This summary figure shows how different combinations of vaccination and lockdown interventions can lead to successful disease control (in terms of minimizing cumulative mortalities) (Figure 5). Even with a low vaccination rate (e.g., ν=0.005), and depending on the severity of lockdown, lockdown durations could be as short as 20 days (for a 80% reduction in β). Alternatively, lockdowns could be of 30 days (for 60% reduction in β) or 50 days (for 40% reduction in β) to achieve successful virus control. However, under this level of vaccination (ν=0.005), it is simply not feasible for a lockdown where β is only reduced by 20% to keep cumulative mortality below a (nominal) 80,000 threshold.

**Figure 6:**
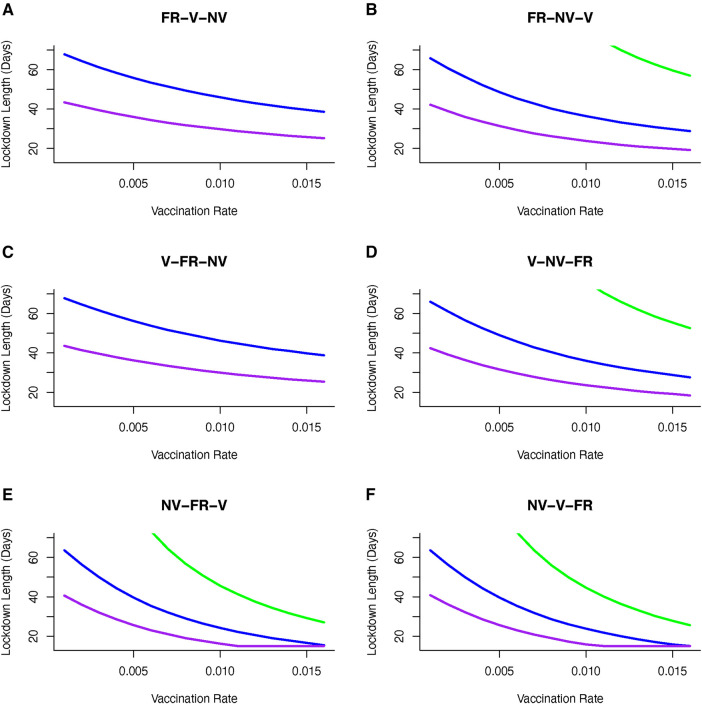
Vaccine sequence to minimize cumulative mortality (below 80K) for varying duration and severity of lockdown and vaccination rate. Sequences are (**A**) Front-line workers (*FR*) then vulnerable (*V*) group then non-vulnerable (*NV*) group, (**B**) Front-line workers (*FR*) then non-vulnerable (*NV*) group then vulnerable (*V*) group, (**C**) Vulnerable (*V*) group then front-line workers (*FR*) then non-vulnerable (*NV*) group, (**D**) Vulnerable (*V*) group then non-vulnerable (*NV*) group then front-line workers (*FR*), (**E**) Non-vulnerable (*NV*) group then front-line workers (*FR*) then vulnerable group, and (**F**) Non-vulnerable (*NV*) group then vulnerable (*V*) group then front-line workers (*FR*). Sequence is to deliver vaccine to first group for 30 days, first and second group from 31–60 days and all groups after 60 days. Only moderately severe (reducing transmission by 60%) or severe (reducing transmission by 80%) achieve optimal control of mortality. Including non-vulnerable group (*NV*) in first or second phase of vaccination achieves better control and can reduce the severity and duration of lockdowns. If front-line workers (*FR*) are first in line for vaccination then the optimal sequence is shown in (**B**). If vulnerable group (*V*) is first in line then to achieve optimal control of mortality requires vaccination of non-vulnerable (*NV*) group before front-line worker (*FR*) group (**D**). [purple line --80% reduction in *β*, blue line --60% reduction in *β*, green line --40% reduction in *β*].

We illustrate how vaccinating different groups in different sequences can influence strategies to achieve the optimal outcome, and most notably impact the time required in lockdown. If a strategy is adopted to vaccinate different groups over sequential 30 day periods (and continuing this vaccination strategy once initiated for each group), then we find that the choice of such longitudinal sequences influences the required lockdown duration and/or severity to achieve optimal disease control. For the majority of sequences, moderate to severe lockdowns are needed to achieve the optimal goal of keeping cumulative mortality below a critical threshold (Figure 6). However, adopting a strategy in which the non-vulnerable group is vaccinated first, followed by the front line worker group followed by the vulnerable group (“NV-FR-V”; Figure 6) allows lockdowns of weaker severity (where disease transmission is reduced by only 20%), albeit these might be of longer duration, in order to achieve the optimal goal.

Importantly, we find that if we consider the sequence of “NV-FR-V,” and select a vaccine rate, then this, represented in Figure 6E, gives us a lockdown length that makes that the vaccination rate optimal. However, of particular interest is that for some transmission reductions e.g., 20%, and for the same vaccination rate, other orders of vaccination are associated with longer lockdowns. Hence there are circumstances where vaccinating non-vulnerable people first may be optimal in terms of minimising time in lockdown.

Apart from the sequence of vaccine delivery, there is again no alternative feasible vaccination strategy where weak lockdowns (transmission is reduced by 20%) that allows mortality levels to be kept below 50,000 people. Targeting the non-vulnerable group in the first or second wave of vaccination allows a broad set of lockdown severity (40%–80% reduction in transmission) strategies to be implemented. If a strategy is adopted to target vulnerable and front line workers then it will require moderate to severe lockdowns (60%–80% reductions in transmission) to achieve the optimal outcome for disease control (Figure 6).

### Vaccination demand: declining vaccine uptake rates

**Figure 7:**
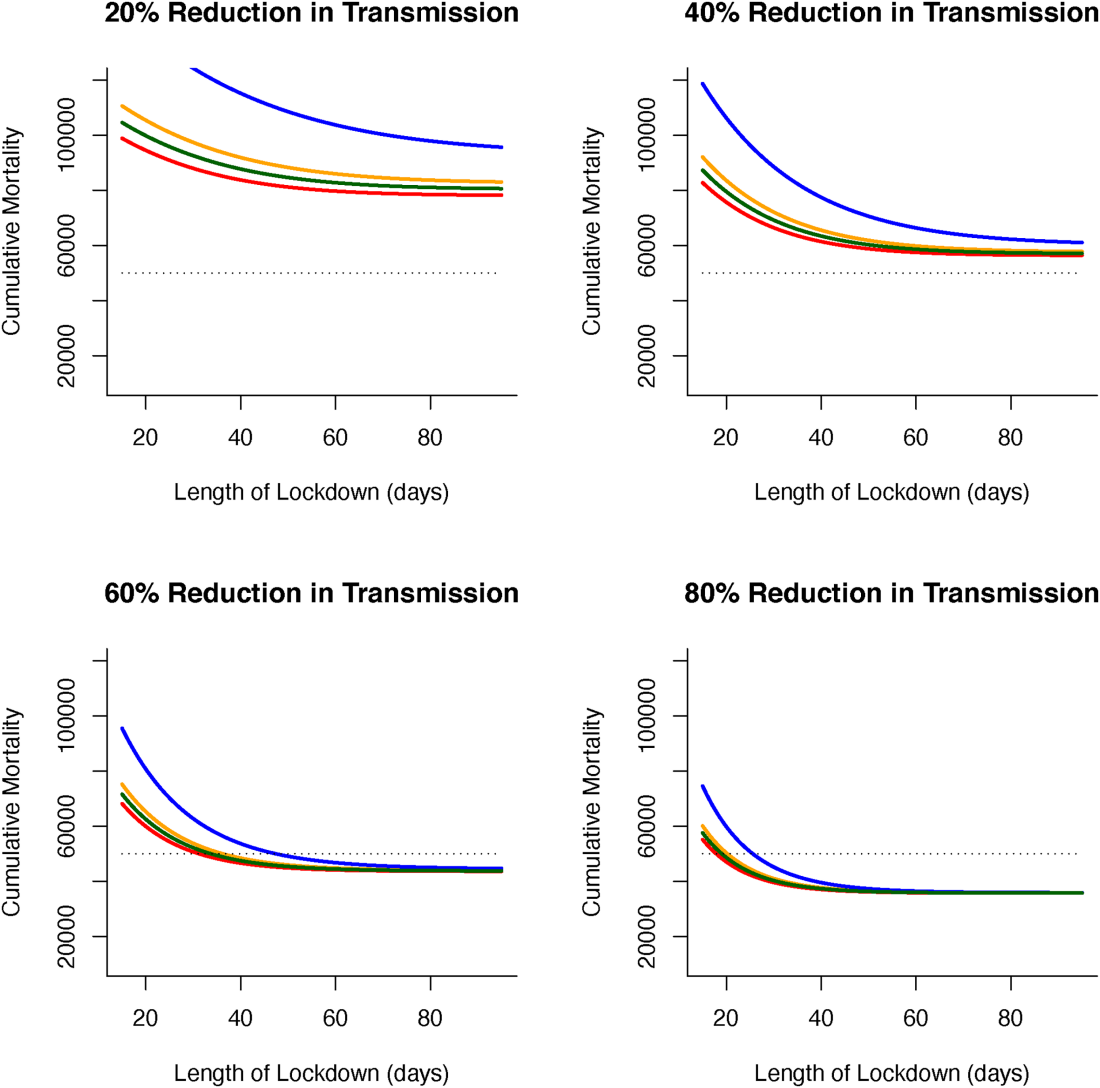
Effects of declining vaccine demand uptake and length of lockdowns (in days) on cumulative virus-induced mortality (dashed line shows 50 K threshold) for different lockdown severities (as reductions in virus transmission during lockdown). Fixed (inelastic) vaccine demand always leads to the lowest levels of cumulative mortality (red line). For declining (elastic) vaccine demand, rapidly (exponential) declining vaccine uptake (blue line) leads to the largest difference from fixed vaccine (compared to linear [(orange line) or polynomial declines (green line)]. The effects of elastic vaccine demand on cumulative mortality can be offset by increasing length (days) and severity (reduction in virus transmission) of lockdowns.

In this final part to our analysis, we illustrate how any changes to vaccine demand and uptake can influence the outcome of disease mitigation and control measures. One particular concern is that as a substantial number of people are vaccinated, and potentially in tandem with initial declines in infection rates, then there will be an emerging complacency and vaccine adoption will fall. However, any (exponentially) declining vaccine uptake is most likely to disrupt control measures and lead to resurgences in disease spread and increases in mortalities (Figure 7). Again, the use of lockdowns of different durations and/or severities can help mitigate against any declining vaccine uptake. Even for weakly restrictive lockdowns (where disease transmission is only reduced by 20%) and if vaccine uptake declines exponentially (here set at $2.0\times 10^{-4}$ per day), we show that long duration lockdowns can reduce cumulative mortality levels (Figure 7). As may be expected, more severe lockdowns, in the event of this sort of loss of vaccine uptake, can limit the required duration of a lockdown and lead to effective disease control.

## Discussion

Here, using mathematical modelling approaches, we have investigated how combining vaccinations and lockdowns can be used to control virus levels (in terms of disease induced levels of mortality). We show that for different levels of vaccination (or vaccine efficacy), lockdowns of different duration and/or severity can be implemented to mitigate levels of mortality. In particular, we highlight that there are combinations of vaccination and lockdowns that can achieve effective virus control. Out of a number of potential strategies for disease control that include promoting natural herd immunity, virus elimination or releasing lockdowns as treatments become available, Sheikh et al. (25) advocated approaches whereby lockdowns were relaxed and virus infection levels managed through contact-tracing strategies. While this might be an achievable endgame, to reach acceptable levels of disease control requires, as we have demonstrated here, combinations of approaches. Rather than relaxing NPIs as a strategy, our analysis suggests that combining them with vaccine programs will constrain levels of mortality. This finding is valid even with low coverage vaccination (or low vaccine efficacy), as lockdowns still allow optimal solutions to be found for mitigating levels of disease.

A non-random distribution of vaccinations may be ineffective even in behaviourally homogeneous populations (26). Moreover, for heterogeneous populations, evaluating the full contact matrix to derive an appropriate vaccination strategy may be impossible. Here, even though we have used the simplifying approximation that within and between contacts are similar ($\beta_{\mathrm{ji}}=\beta$), the force of infection ($\chi_{i}$) differs between groups and, as such, the non-random distribution of vaccines can be inefficient at achieving sufficient coverage for the disease to fade out. Similar findings for age-structured models (27) and vaccine sharing strategies (28) corroborate this finding. Here, this inefficiency in vaccination occurs due to population size differences between the groups and the relative differences that this has on both the force of infection and mitigating the levels of disease-induced mortality within different groups. In particular, we find that vaccinating individuals in the vulnerable group (a relatively small group) should be rolled out together with mass vaccination across the larger non-vulnerable group to achieve necessary public health benefits of herd immunity and reducing mortality.

These sorts of well-planned vaccination strategies may lead to spatial and temporal clusterings. These groupings and others such as social clusterings generate further levels of heterogeneity that are likely to make achieving optimal outcomes based on vaccination strategies alone challenging. In addition, groups that eschew vaccinations may make disease control more difficult. In fact, any “clustering of exemptions” necessitates greater vigilance around the emergence of a “critical mass” where individual decisions to decline vaccination impinge on the collective (public health) benefit and restricts vaccine coverage (29–31). Here, we broadly account for these factors, and consider explicitly declining vaccination uptake should groups decide to alter behaviours during mass-roll out of the vaccine. As expected, a rapid (exponential) decline of vaccine uptake is most precipitous in terms of optimal disease control outcomes. Our results confirm that lockdowns and other non-pharmaceutical interventions can be used to mitigate against the effects of social clusterings and loss of vaccine uptake/efficacies. However, understanding the way in which these groupings form will be critical to determine how robust, in terms of severity and duration, lockdowns would need to be to achieve optimal disease control outcomes for ongoing disease outbreaks.

Optimal control approaches have been widely used in understanding the control of infectious diseases and there have been several applications to understanding the SARS-CoV-2 pandemic. For instance, our own work (13, 28, 32) has, respectively, focused on the use of optimal control approaches to understand how the use of NPIs and lockdowns could be eased and yet still minimize hospitalizations, how circuit-breakers could be optimally used to disrupt epidemic peaks, and the optimal approaches to sharing vaccines between nations. Other studies have focused on the use of optimal control approaches for mitigating disease mortalities and the use of NPIs (33) and how vaccinations could be administered to minimize mortalities (34).

Perkins & Espana (33) use COVID-19 epidemic data from the USA to parameterise an unstructured SEIR framework, although with additional asymptomatic, hospitalised and vaccinated groups to investigate the optimal use of NPIs. In their formulation, only susceptible individuals received the vaccine and optimal solutions then focused on minimizing both use of NPIs as a mechanism to reduce disease transmission) and deaths. Perkins & Espana (2020) find that relaxing NPIs too soon can have major implications for longer term disease control, and that maintaining stricter levels of control minimizes deaths. Here, our results support this finding that the severity of control (i.e., as expressed as reductions in disease transmission β) can mitigate levels of disease-induced deaths. Our main finding is that in addition, with optimal strategies established for vaccination, this supports the minimisation of deaths and can, under certain conditions, offset the need for severe or long lasting NPIs.

Libotte et al. (34) also use a SIR model combined with optimal control approaches to investigate vaccine delivery strategies. In their study, the objective functional is focused to minimize the number of infected individuals and the total doses of vaccine required. Their use of an inequality constraint is used to model limitations related to vaccine availability and production. With the choice of linear terms in the objective functional, the optimal solutions are on-off (“bang-bang”) control with variable time between delivery of vaccines to minimize the number of infections. Our results contrast with this bang-bang control. This highlights the effects of different uses of cost structures. In particular, our differences will be due to using a quadratic for increasing costs to capture difficulties in achieving vaccination targets as the number of vaccinated individuals increases. Differences are also due to the application of the optimal control problem to a structured (rather than unstructured) population. Our key result is that the sequence of vaccine delivery to different groups is critical to achieving disease control and minimizing the public health burden of disease-induced deaths.

As noted, neither of these previous studies (33, 34) consider population structure and the interaction between vaccines and NPIs as concomitant approaches to disease control and minimizing disease induced deaths. We argue that these sort of optimal control approaches provide a “weight of evidence” for more pluralistic approaches to controlling the infection, and especially as appropriate constraints can be included in solving numerical optimal models of the epidemiological dynamics.

As vaccines for SARS-CoV-2 infections were rolled out, and countries thereby implemented mass inoculation programs, many places experienced further waves of infection. This required a difficult balance between providing the benefits of vaccination programs as a route to returning to normality, while still requiring the continued use of NPIs (such as strict lockdown rules) in the interim. For this reason, our analysis highlights, that for future puclic health planning, it is timely to investigate a broad range and combination of options available to minimise the use of NPIs in parallel with emerging vaccination programmes.

Here, we have used a mathematical model to investigate the effect of lockdowns together with mass vaccination plans. For the three quantities of different rates of vaccinations, extent of restrictions that impacts virus transmission and number of deaths that will protect health services, we derive the shortest lockdown length. Our mathematics of optimisation determines the shortest lockdown time for these three quantities, and critically, across different options of which groups of people to vaccinate first. We use three discrete groups of people, of vulnerable, front-line workers and non-vulnerable. As an additional component to our numerical calculations, we also allow for “vaccine elasticity,” where individuals may become less concerned about receiving a vaccine as disease infection rates fall. We also allow consideration of vaccines that are not fully efficacious as we evaluate optimal outcomes in terms of shortest lockdowns for prescribed maximum levels of mortality. We argue that our use of appropriately developed structured epidemiological models provides a robust way to investigate these epidemiological outcomes. Our optimal control approaches allow the best combinations (here for parameter constraints applicable to the UK) to be determined. However, these outcomes are parameter-dependent, and might change across different locations, temporal scales, vaccine efficacies and/or SARS-CoV-2 strain dependencies.

Our headline findings are as follows. As might be expected, to require relatively short lockdowns, NPIs have to be sufficiently severe as to suppress transmission. Less effective vaccines imply longer lockdowns, as does a larger vaccine elasticity. However, to achieve appropriate levels of disease control and contrary to an approach focusing on vaccinating the vulnerable group first, we find that the optimal vaccination policy is to inoculate the larger (non-vulnerable) demographic group first, then followed by front-line workers and then the vulnerable. This finding might at first appear counter-intuitive, given the order-of-magnitude difference in death rate for those encouraged to shield against COVID-19 (i.e., in the vulnerable category). The reason for this finding is our analysis assumes that the time required to vaccinate the vulnerable group is identical to that of the much larger non-vulnerable group. As the non-vulnerable group is much larger, many more people can be vaccinated per day under that assumption, causing the disease to decline more quickly and yet still constraining the overall number of deaths.

Our expectation is that this analysis will encourage additional theoretical and empirically validated studies to understand further how aspects of heterogeneity, demographic, either by group or age structure, or geographic, either by location or within/outside lockdowns impacts the epidemiological dynamics and outcomes of virus spread. For instance, one aspect of our analysis not considered is the differentiation between the vaccination of individuals in lockdown and those not in lockdown. This heterogeneity, together the demographic effects of age or group structure is likely to have important implications for the epidemiology and virus dynamics, and developing public health control interventions. Furthermore, analyses could include the associated immediate effects of lowered transmission by lockdowns, both within and between different groups, as well as a more detailed understanding of differential effects of vaccinated individuals in and outwith lockdowns, the impact of choice of vaccination order on required lockdown lengths and neutralizing/non-neutralizing effects of vaccination in preventing onward disease transmission. Of all of these possibilities, in particular it is the impact of the choice of vaccination order between groups on required lockdown lengths that we believe remains to be explored further and is therefore worthy of substantial further investigation.

## Data Availability

The datasets presented in this study can be found in online repositories. The names of the repository/repositories and accession number(s) can be found below: https://osf.io/xvunt/.

## Appendix

### Optimal control

Here, the aim is to find an optimal way to control infection (by minimizing disease-induced mortality) through an epidemic under different transmission rates and increasing difficulty of vaccination delivery as the number of vaccinated individuals increases.

#### Unstructured model

We begin by introducing the governing equations (see main text for further details) We use an S-I-R-V framework to describe the epidemiological dynamics. The dynamics for susceptible individuals follow

$$\frac{dS}{dt}=-\frac{\beta S(t)I(t)}{N(t)}+\sigma R(t)-\mu S(t)-\nu (t)S(t)$$

The dynamics for infected individuals follow:

$$\frac{dI}{dt}=\frac{\beta S(t-\tau )I(t-\tau )}{N(t-\tau )}\exp(-\mu \tau )-\left(\alpha +\mu +\gamma \right)I(t)$$

The dynamics for recovered individuals follow:

$$\frac{dR}{dt}=\gamma I(t)-\left(\nu (t)+\mu +\sigma \right)R(t)$$

and the dynamics for the total number of vaccinated individuals are:

$$\frac{dV}{dt}=\nu (t)(R(t)+S(t))-\mu V(t)$$

where β is the disease transmission rate, σ the loss of immunity, μ is the background death rate, ν(t) is the vaccination rate (on both susceptible and recovered individuals), τ is the incubation window, α is the disease induced death rate, γ is the disease recovery rate and N(t)=S(t)+I(t)+R(t)+V(t).

The objective functional is defined in terms of the rate of vaccination, the number of individuals vaccinated and level of disease induced mortality such that during the epidemic of time length T the “costs” of vaccination increase. The goal is to minimize these “costs” of vaccination and keep daily disease-induced mortality below a threshold, together with a terminal condition (h[I(T),T]) accounting for ongoing “cost” of infection:

$$\underset{0<\nu \leq 1}{\min(J[x,\;\nu ])}=\underset{0<\nu \leq 1}{\min}\left(h[I(T),\;T]+\int_{0}^{T}\left[\frac{\nu^{2}(t)}{2}V(t)+\exp(\alpha I(t)-Z)\rho \right]dt\right)$$

where the control is vaccination rate (ν(t)):, T is the length of the disease epidemic wave and Z is the critical level of daily disease induced mortality that can not be exceeded.

To solve the optimization problem, a Hamiltonian operator (H) and adjoint system (see below) are formed. As with all optimal control problems, the Hamiltonian operator is formed as H=f(t,x,ν)+λg(t,x,ν), where f represents the ’cost’ function, g represents the governing equations (through time t for dynamical system x and control ν), and λ is a multipler function (Kamien & Schwartz 2012). For the unstructured disease model, the Hamiltonian operator is:

$$\begin{array}{rl} H & =\frac{1}{2}\nu (t{)}^{2}V(t)+\exp(\alpha I(t)-Z)\rho \\ & \;+\lambda_{1}\left(-\frac{\beta S(t)I(t)}{N(t)}+\sigma R(t)-\mu S(t)-\nu S(t)\right)\\ & \;+\lambda_{2}\left(\frac{\beta S(t-\tau )I(t-\tau )}{N(t-\tau )}exp(-\mu \tau )-\left(\alpha +\mu +\gamma \right)I(t)\right)\\ & \;+\lambda_{3}\left(\gamma I(t)-\left(\nu +\mu +\sigma \right)R(t)\right)\\ & \;+\lambda_{4}\left(\nu (R(t)+S(t))-\mu V(t)\right). \end{array}$$

Expressions for the characterization of the control are derived from minimizing the Hamiltonian operator. Each adjoint variable ($\lambda_{i}$) satisfies an equation found by differentiating the Hamiltonian operator with respect to the corresponding state variable, and then negating this derivative:

$$\begin{array}{rl} \frac{d\lambda_{1}}{dt}=-\frac{dH}{dS} & =\lambda_{1}\left(\mu +\nu \right)-\lambda_{1}\left[\frac{\beta SI}{(S+I+R+V{)}^{2}}-\frac{\beta I}{\left(S+I+R+V\right)}\right]\\ & \;-\lambda_{2}\left[-\frac{\beta SI}{(S+I+R+V{)}^{2}}+\frac{\beta I}{\left(S+I+R+V\right)}\right]-\lambda_{4}\nu \end{array}$$

$$\begin{array}{rl} \frac{d\lambda_{2}}{dt}=-\frac{dH}{dI} & =-\exp(\alpha I-Z)\rho \alpha -\lambda_{1}\left[\frac{\beta SI}{(S+I+R+V{)}^{2}}-\frac{\beta S}{\left(S+I+R+V\right)}\right]\\ & \;-\lambda_{2}\left[-\frac{\beta SI}{(S+I+R+V{)}^{2}}+\frac{\beta S}{\left(S+I+R+V\right)}\right]+\lambda_{2}\left(\alpha +\mu +\gamma \right)-\lambda_{3}\gamma \end{array}$$

$$\begin{array}{rl} \frac{d\lambda_{3}}{dt}=-\frac{dH}{dR} & =-\lambda_{1}\sigma -\lambda_{1}\left(\frac{\beta SI}{(S+I+R+V{)}^{2}}\right)-\lambda_{2}\left(-\frac{\beta SI}{(S+I+R+V{)}^{2}}\right)\\ & \;+\lambda_{3}\left(\nu +\mu +\sigma \right)-\lambda_{4}\nu \end{array}$$

$$\begin{array}{rl} \frac{d\lambda_{4}}{dt}=-\frac{dH}{dV} & =-\lambda_{1}\left(\frac{\beta SI}{(S+I+R+V{)}^{2}}\right)-\lambda_{2}\left(-\frac{\beta SI}{(S+I+R+V{)}^{2}}\right)+\lambda_{4}\mu -\frac{1}{2}\nu^{2} \end{array}$$

With appropriate boundary conditions (for both the initial and the terminal conditions) , the optimal control (ν) then minimizes the Hamiltonian operator such that $\frac{\partial H}{\partial \nu }=0$ for $\nu =\nu^{*}$:

$$\frac{\partial H}{\partial \nu }=\nu V-\lambda_{1}S-\lambda_{3}R+\lambda_{4}(R+S).$$

where $\nu^{*}$ is:

$$\nu^{*}=\frac{\lambda_{1}S+\lambda_{3}R-\lambda_{4}(R+S)}{V}$$

The second derivative of H indicates the solution is a minimum as:

$$\frac{\partial^{2}H}{\partial \nu^{2}}=V.$$

This is positive when V>0, so solutions are determined as the number of vaccinated individuals increases.

#### Structured model

The optimal control approach can be extended to the group-structured epidemiological model. The aim remains to find the most optimal way to control infection by minimizing disease induced mortality, costs of vaccination and ongoing infection costs.

The governing dynamics, by extension, follow a similar framework to the unstructured model. The dynamics for the $i^{th}$ group are described by:

$$\begin{array}{rl} \frac{dS_{i}}{dt} & =-\chi_{i}S_{i}(t)+\sigma R_{i}(t)-\mu S_{i}(t)-\nu_{i}S_{i}(t),\;\\ \frac{dI_{i}}{dt} & =\chi_{i}(t-\tau )S_{i}(t-\tau )exp(-\mu \tau )-\left(\alpha_{i}+\mu +\gamma_{i}\right)I_{i}(t),\;\\ \frac{dR_{i}}{dt} & =\gamma_{i}I_{i}(t)-R_{i}(t)\left(\nu_{i}+\mu +\sigma \right),\;\\ \frac{dV_{i}}{dt} & =\nu_{i}\left(R_{i}(t)+S_{i}(t)\right)-\mu V_{i}(t),\; \end{array}$$

where $N_{i}(t)=S_{i}(t)+I_{i}(t)+R_{i}(t)+V_{i}(t)$ and $\chi_{ij}$ is the force of infection $\chi_{i}=\sum_{\;j}\beta_{i,j}\frac{I_{j}}{N_{j}}$. For simplicity $\beta_{ij}=\beta_{i}$ such that there is a single fixed transmission rate both between and within a group which can differ between groups. Other parameters are the vaccination rate ($\nu_{i}$). recover rate ($\gamma_{i}$) and disease-induced death rate ($\alpha_{i}$) associated with the $i^{th}$ group whereas μ (background death rate) and σ (loss of immunity rate) are common to all groups.

The objective functional is now defined in terms of the rate of vaccination, number of individuals vaccination, levels of disease induced mortality and ongoing costs of infections for *each group*:

$$\underset{0<\nu \leq 1}{\min(J[x,\;\nu ])}=\underset{0<\nu \leq 1}{\min}(\sum_{i}h[I_{i}(T),\;T]+\int_{0}^{T}\sum_{i}\left[\frac{\nu_{i}^{2}(t)}{2}V_{i}(t)+\exp(\alpha_{i}I_{i}(t)-Z_{i})\rho \right]dt)$$

where the control is now the group specific vaccination rate ($\nu_{i}$) and $Z_{i}$ is the critical level of disease induced mortality that can not be exceed for each group.

The Hamilton operator is of the form $H=\sum_{i}f_{i}(t,\;x_{i},\;\nu_{i})+\lambda_{i}g_{i}(t,\;x_{i},\;\nu_{i})$ where $f_{i}$ represents the cost functions and $g_{i}$ the governing dynamics, and $\lambda_{i}$ is a multiplier function, for each of the groups.

$$\begin{array}{rl} H & =\sum_{i}\frac{1}{2}\nu_{i}(t{)}^{2}V(t)+\exp(\alpha_{i}I_{i}(t)-Z)\rho \\ & \;+\lambda_{1i}\left(-\chi_{i}S_{i}(t)+\sigma R_{i}(t)-\mu S_{i}(t)-\nu_{i}S_{i}(t)\right)\\ & \;+\lambda_{2i}\left(\chi_{i}(t-\tau )S_{i}(t-\tau )\exp(-\mu \tau )-\left(\alpha_{i}+\mu +\gamma_{i}\right)I_{i}(t)\right)\\ & \;+\lambda_{3}\left(\gamma_{i}I_{i}(t)-\left(\nu_{i}+\mu +\sigma \right)R_{i}(t)\right)\\ & \;+\lambda_{4}\left(\nu_{i}(R_{i}(t)+S_{i}(t))-\mu V_{i}(t)\right). \end{array}$$

Explict expression for the multipliers ($\lambda_{yi}$) are found by differentiating this operator with respect to the corresponding state variable and then negating the derivative:

$$\begin{array}{rl} \frac{d\lambda_{1i}}{dt}=-\frac{dH}{dS_{i}} & =\lambda_{1i}\left(\mu +\nu_{i}\right)-\lambda_{1i}\left[\frac{\beta_{i}S_{i}I_{i}}{(S_{i}+I_{i}+R_{i}+V_{i}{)}^{2}}-\frac{\beta_{i}I_{i}}{\left(S_{i}+I_{i}+R_{i}+V-i\right)}\right]\\ & \;+\lambda_{1i}\sum_{j}\frac{\beta_{j}I_{j}}{N_{j}}-\lambda_{2i}\left[-\frac{\beta_{i}S_{i}I_{i}}{(S_{i}+I_{i}+R_{i}+V_{i}{)}^{2}}+\frac{\beta_{i}I_{i}}{\left(S_{i}+I_{i}+R_{i}+V_{i}\right)}\right]\\ & \;-\lambda_{2i}\sum_{j}\frac{\beta_{j}I_{j}}{N_{j}}-\lambda_{4}\nu_{i} \end{array}$$

$$\begin{array}{rl} \frac{d\lambda_{2i}}{dt}=-\frac{dH}{dI_{i}} & =-\exp(\alpha_{i}I_{i}-Z_{i})\rho \alpha_{i}-\lambda_{1i}\sum_{\;j=1}^{3}\left[\frac{\beta_{i}S_{j}I_{i}}{(S_{i}+I_{i}+R_{i}+V_{i}{)}^{2}}-\frac{\beta_{i}S_{j}}{\left(S_{i}+I_{i}+R_{i}+V_{i}\right)}\right]\\ & \;-\lambda_{2i}\sum_{\;j=1}^{3}\left[-\frac{\beta_{i}S_{j}I_{i}}{(S_{i}+I_{i}+R_{i}+V_{i}{)}^{2}}+\frac{\beta_{i}S_{j}}{\left(S_{i}+I_{i}+R_{i}+V_{i}\right)}\right]+\lambda_{2i}\left(\alpha_{i}+\mu +\gamma_{i}\right)-\lambda_{3i}\gamma_{i} \end{array}$$

$$\begin{array}{rl} \frac{d\lambda_{3i}}{dt}=-\frac{dH}{dR_{i}} & =-\lambda_{1i}\sigma -\sum_{\;j=1}^{3}\lambda_{1j}\left(\frac{\beta_{i}S_{j}I_{i}}{(S_{i}+I_{i}+R_{i}+V_{i}{)}^{2}}\right)-\sum_{\;j=1}^{3}\lambda_{2j}\left(-\frac{\beta_{i}S_{j}I_{i}}{(S_{i}+I_{i}+R_{i}+V_{i}{)}^{2}}\right)\\ & \;+\lambda_{3i}\left(\nu_{i}+\mu +\sigma \right)-\lambda_{4}\nu_{i} \end{array}$$

$$\begin{array}{rl} \frac{d\lambda_{4i}}{dt}=-\frac{dH}{dV_{i}} & =-\sum_{\;j=1}^{3}\lambda_{1j}\left(\frac{\beta_{i}S_{j}I_{i}}{(S_{i}+I_{i}+R_{i}+V_{i}{)}^{2}}\right)-\sum_{\;j=1}^{3}\lambda_{2j}\left(-\frac{\beta_{i}S_{j}I_{i}}{(S_{i}+I_{i}+R_{i}+V_{i}{)}^{2}}\right)\\ & \;+\lambda_{4}\mu -\frac{1}{2}\nu_{i}^{2} \end{array}$$

Expressions for optimal vaccination rates ($\nu_{i}$) for each group are found from minimizing this Hamiltonian operator such that $\frac{dH}{d\nu_{i}}=0$ for $\nu_{i}=\nu_{i}^{*}$:

$$\frac{dH}{d\nu_{i}}=\nu_{i}V_{i}-\lambda_{1i}S_{i}-\lambda_{3i}R_{i}+\lambda_{4i}(R_{i}+S_{i})$$

So $\nu_{i}^{*}$ is given by:

$$\nu_{i}^{*}=\frac{\lambda_{1i}S_{i}+\lambda_{3i}R_{i}-\lambda_{4i}(R_{i}+S_{i})}{V_{i}}$$

which is a minimum as $\frac{\partial^{2}H}{\partial \nu^{2}}>0$.
